# Decentralized Data Sharing of Tissue Microarrays for Investigative Research in Oncology

**Published:** 2007-06-06

**Authors:** Wenjin Chen, Cristina Schmidt, Manish Parashar, Michael Reiss, David J. Foran

**Affiliations:** 1 Center for Biomedical Imaging & Informatics, UMDNJ–Robert Wood Johnson Medical School; 2 The Applied Systems Software Laboratory, Department of Electrical and Computer Engineering, Rutgers University; 3 The Cancer Institute of New Jersey, UMDNJ–Robert Wood Johnson Medical School

**Keywords:** Tissue microarray, Tissue microarray analysis, Data Sharing, Content-based Search, Distributed Hash Table

## Abstract

Tissue microarray technology (TMA) is a relatively new approach for efficiently and economically assessing protein and gene expression across large ensembles of tissue specimens. Tissue microarray technology holds great potential for reducing the time and cost associated with conducting research in tissue banking, proteomics, and outcome studies. However, the sheer volume of images and other data generated from even limited studies involving tissue microarrays quickly approach the processing capacity and resources of a division or department. This challenge is compounded by the fact that large-scale projects in several areas of modern research rely upon multi-institutional efforts in which investigators and resources are spread out over multiple campuses, cities, and states. To address some of the data management issues several leading institutions have begun to develop their own “in-house” systems, independently, but such data will be only minimally useful if it isn’t accessible to others in the scientific community. Investigators at different institutions studying the same or related disorders might benefit from the synergy of sharing results. To facilitate sharing of TMA data across different database implementations, the Technical Standards Committee of the Association for Pathology Informatics organized workshops in efforts to establish a standardized TMA data exchange specification. The focus of our research does not relate to the establishment of standards for exchange, but rather builds on these efforts and concentrates on the design, development and deployment of a decentralized collaboratory for the unsupervised characterization, and seamless and secure discovery and sharing of TMA data. Specifically, we present a self-organizing, peer-to-peer indexing and discovery infrastructure for quantitatively assessing digitized TMA’s. The system utilizes a novel, optimized decentralized search engine that supports flexible querying, while guaranteeing that once information has been stored in the system, it will be found with bounded costs.

## Introduction

Tissue microarray (TMA) technique enables researchers to extract small cylinders of tissue from histological sections and arrange them in a matrix configuration on a recipient paraffin block such that hundreds can be analyzed simultaneously (Kononen et al. 1998; Rimm et al. 2001; Braunschweig et al. 2004). This new technique should not be confused with DNA microarrays, wherein each tiny spot on the grid represents a unique cloned complementary DNA (cDNA) or oligonucleotide. During the period of 1998 to 2001, a great number of research groups contributed in the process in which TMA technology was validated and tested extensively for use in cancer research. As a result, it is now generally accepted that two to four samples taken from different regions of each donor tissue block provide sufficient morphologic information for a reliable evaluation of the specimen to be rendered. These validation works were carried out in a wide range of diseases by comparing TMA analysis with whole tissue sections or by validating results with cDNA microarray findings (Camp et al. 2000; Gulmann et al. 2003; Mucci et al. 2000b; Fernebro et al. 2002; Hedvat et al. 2002; Natkunam et al. 2001; Engellau et al. 2001; Moch et al. 1999; Nocito et al. 2001; Sallinen et al. 2000; Pacifico et al. 2004; Beer et al. 2002). Some investigators refined and tuned the array preparation protocols in order to accommodate specific types of specimens, e.g. cell lines, or to improve the reliability of the method (Andersen et al. 2001; Chung et al. 2002; Dan et al. 2004; DiVito et al. 2004; Fejzo and Slamon, 2001; Kylaniemi et al. 2004; Matysiak et al. 2003).

One of the advantages of TMA technology is that it serves to amplify limited tissue resources by generating large numbers of small core biopsies, rather than a single section. Another attractive feature of the TMA technique is that specimens from different donor tissue blocks are treated in an identical manner in terms of incubation times, temperatures and washing conditions making comparison of expression among the discs comprising a given array feasible. Currently, the primary methods used to evaluate the arrays involve manual, interactive review of TMA samples under the microscope while they are subjectively evaluated and scored. An alternate, but less utilized strategy is to sequentially and manually digitize specimens for subsequent semi-quantitative assessment (Mucci et al. 2000a; Matysiak et al. 2003). Both procedures ultimately involve the interactive evaluation of TMA samples which is a slow, tedious process that is prone to error. It was recently reported that to help automate the process of characterizing the staining intensities of tissue samples, the AQUA (Automated Quantitative Analysis) system was developed. The system is a molecular based approach for quantitatively assessing protein expression. One of the chief motivations for developing the system was to reduce the degree of inter- and intra-observer variability associated with pathologist-based evaluation of samples (Rubin et al. 2004). Several other groups have undertaken projects to read immunohistochemistry (IHC) TMA specimen using commercial cDNA microarray readers (Haedicke et al. 2003; Rao et al. 2002). Since about 2001 the idea of developing reliable and effective methods and protocols to quantitative IHC TMA analysis has become an extremely active area of research (Ayala et al. 2003; Camp et al. 2002; Camp et al. 2003). It is expected that by reducing the amount of time and effort to process TMA’s, these new technologies may serve to accelerate the pace of research in cancer biology, drug discovery, and oncology.

Two recent studies highlight this point. In a recent study on HER2 expression in breast cancer tissue, it was found through the use of TMA’s that higher levels of HER2 protein correlated with poorer clinical outcomes (Camp et al. 2003). In an earlier report, the same team studied the prognostic value of beta-catenin expression in 310 colon carcinoma specimens which had been collected between 1971 and 1982 (Camp et al. 2002). When the team analyzed these tissue sections using the traditional four-point scale they saw no correlation between the amount of nuclear beta-catenin and clinical prognosis. But when the group stratified the differing amounts of expression among the samples using a continuous 1,000 point scale they found that the tissue cores in the top 10% of nuclear beta-catenin expression correlated with significantly worse prognosis.

Tissue microarray technology holds tremendous potential for reducing the time and cost associated with conducting research in tissue banking, proteomics, and outcome studies, however, analyzing, sharing, and managing the data that TMA’s generate creates a number of significant challenges. To address some of the data management issues several leading institutions have begun to develop their own functional systems “in-house”, but such data will be only minimally useful if it isn’t accessible to others in the scientific community. Researchers at different institutions studying the same or related diseases might potentially benefit from the synergy of sharing results. The Technical Standards Committee of the Association for Pathology Informatics is working to establish a TMA data exchange specification which is free and non-proprietary (Berman et al. 2003; Berman et al. 2004). In spite of some of the progress that has been made toward automating array production and standardizing data for exchange, however, the promise of TMAs remains partly unfulfilled because of the lack of quick, reliable methods for performing *unsupervised* quantitative analysis.

At the same time, there exists a real need for reliable tools which enable individuals to dynamically acquire, share and assess imaged specimens and correlated data. The focus of our research is not on the establishment of standards for exchange, but rather builds on these efforts and concentrates on the design, development and deployment of a decentralized collaboratory for the unsupervised characterization and seamless and secure discovery and sharing of TMA data. Specifically, we present a self-organizing, peer-to-peer indexing and discovery infrastructure for quantitatively assessing digitized TMA’s.

The rich diversity and large volumes of TMA data that makes indexing, cataloging and sharing non-trivial and renders centralized solutions infeasible. Today, TMAs can contain from tens to hundreds of samples (0.6 to 2mm in diameter) arranged on a single slide. A digitized TMA specimen containing just 400 discs can easily approach 18GB in size. Given the increasing number of institutions and investigators utilizing TMA technology it is likely that modern facilities may easily generate tens of thousands of entries and terabytes of data. Clearly archiving, indexing and cataloging and mining this data across the TMA research community is a significant challenge. Further, the increasing popularity of TMA has lead to more and more medical and research institutions being interested and conducting research in this area. While the exact focus of the research conducted by each of these groups may differ in terms of the patient group, the type of cancer, and/or the nature of the staining, being able to share data and meta-data has many advantages. Sharing experimental results and clinical outcomes data could lead to huge benefits in drug discovery and therapy planning. While some leading institutions are developing data management systems for TMA data, these systems are only minimally useful if the data isn’t accessible to others in the scientific community. However, the size of the data involved as well as issues of ownership can quickly limit the scalability and feasibility of this approach.

This paper presents the design, development and evaluation of a prototype peer-to-peer collaboratory for imaging, analyzing, and seamlessly sharing tissue microarrays (TMA), correlated clinical data, and experimental results across a consortium of distributed clinical and research sites. Key components of the collaboratory addressed in this paper include:

### Specification of Semantic Metadata Schematics for TMA

A key requirement for effective sharing of TMA data and metadata is the definition of semantic schemas for describing the TMA sample, the patient parameters, the evaluations conducted and the observed results. We propose an XML schema that builds on emerging metadata standards and is sufficiently rich to capture these dimensions and can be effectively parsed and presented using conventional technologies.

### Mechanisms and Tools for Automated TMA Analysis

As mentioned above, current procedures for TMA analysis ultimately involve the interactive evaluation of TMA samples which is a slow, tedious process that is prone to error. Recent studies showed that having a pathologist score the specimens yields results that are subjective, difficult to reproduce, and do not reflect subtleties. Reliable quantitative measurements will allow investigators to make accurate predictions about patient outcomes and response to therapy. But for the most part, the promise of TMAs remains unrealized because scientists lack methods of high throughput, automated quantitative evaluation.

We have already reported the development of a web-based prototype for automatically imaging, analyzing, and archiving tissue microarrays. The software is both platform- and operating system-independent and with minor modifications to the configuration file can interface with any commercially available robotic microscopy equipment. The software is developed with a modular design to facilitate future integration with automatic slide scanners as well. The system utilizes a combination of sophisticated image processing and pattern recognition strategies to co-register specimens while the software directs a robotic microscope to systematically image specimens at multiple optical magnifications, extract spectral and spatial signatures of the specimen and populate local or distributed relational databases with the resulting data including pointers to imaged arrays. The prototype features both stand-alone and network modes. A visually intuitive interface was developed to enable local and remote users to manipulate the digitized arrays in order to facilitate the organization of specimens for new experiments and to provide a means for data assimilation (Chen et al. 2002).

### Peer-to-Peer Infrastructure for Indexing and Discovery of TMA Data and Metadata

In addition to the algorithmic and software development that is required for analyzing tissue microarrays, reliable tools are also needed to enable individual groups to dynamically acquire and seamlessly share imaged specimens and correlated metadata. However scalable information discovery in the absence of global knowledge of naming conventions remains a fundamental problem in large, decentralized, distributed environments. This is due to the heterogeneous nature and large volume of data and resources, their dynamism and the dynamism of the sharing environment. As a result, an information indexing and discovery system has to be efficient, fault-tolerant and self-organizing. Further, in the case of TMA data, security as well as the ability of each research group to maintain ownership as well as access control capabilities to their data is critical.

As a part of the TMA collaboratory we present Squid, a P2P information indexing and discovery infrastructure. Each peer (e.g. research institution) in this system maintains ownership of its data and only publishes (in a controlled manner) metadata describing its data, which can then be discovered and search externally. The key innovation is a dimension reducing indexing scheme that effectively maps the multidimensional metadata information space to physical peers. Note that access to TMA data in this system is always controlled by the owner of the data.

### Flexible Query Engine with Search Guarantees

A key requirement for the TMA collaboratory is the ability to flexibly and efficiently search TMA data and metadata across peer site using keywords, partial keywords, wildcards and ranges. Further, the underlying query engine should guarantee that all existing data elements that match a query are found with bounded costs. The Squid query engine supports such complex queries and guarantees that all existing data elements that match a query will be found with bounded costs in terms of number of messages and number of nodes involved.

The grid-like organization of tissue microarrays lends itself to quantitative analysis. Although some DNA microarray readers are capable of reading tissue array slides, automatic imaging and evaluation of tissue microarray samples presents several technical challenges. For example, the content of each well on a cDNA microarray chip can generally be considered homogeneous, and hence it is sufficient to describe the expression outcomes by numerical expression levels, which can be generated by relatively straightforward image processing protocols. On the contrary, tissue microarrays are quite heterogeneous in their composition. Depending on the type of tumor or tissue section analyzed, the area of interest may represent nearly the entire disc or only a small percentage thereof. For example, a pancreatic carcinoma or lobular carcinoma of the breast with substantial desmoplastic response may show stromal tissue representing a large percentage of the total area of the disc. If the goal of the assay is to determine epithelial cell expression of a given marker, a protocol must be used that evaluates only that region of the disc. The protocol must not only be able to select the region of interest but also be able to normalize it so that the expression level read from any given disc can be compared with that of other discs.

Our group has made significant progress towards performing unsupervised quantitation of protein expression. Proof of concept of the color decomposition approach using breast cancer tissue array stained with anti-Smad antibodies was recently described (Chen et al. 2004). A new active contour snake based upon robust estimation and a color gradient model has also been developed and reported (Yang et al. 2004; Yang et al. 2005). These advancements now make it possible to investigate the use of these algorithms for a broader range of cancer arrays and to consider the next level of complexity i.e. differential cell analysis and sub-cellular localization.

## Methods

### Imaging and data warehousing

Imaging of the TMAs benefit from the development of a Computer-Assisted Microscopy module (CAM). One of the features of CAM is an auto-focusing module which was developed to enable scanning and imaging processes to operate in *unsupervised* fashion. The auto-focusing protocol utilizes Shannon’s entropy (Cover and Thomas, 1991) as detailed below. Given that *N* is the number of pixels in the image and *P_i_* is the value at pixel *i* (1 ≤ *i* ≤ *N* ), the probability that a photon falls into pixel *i* is computed as,

$$p_{i}=\frac{\#\;photons\;in\;pixel\;i}{\#\;photons\;in\;entire\;image}=\frac{P_{i}}{\sum_{j=1}^{N}P_{j}},$$

and image entropy is defined as,

$$S=-\sum_{i=1}^{N}p_{i}\text{log}(p_{i})=\sum_{i=1}^{N}p_{i}\text{log}\left(\frac{1}{p_{i}}\right)$$

Of all possible images of *N* pixels, a uniform image, which we consider completely out-of-focus, has maximum entropy, *S*_0_ = log(*N*), where *S*_0_ is solely dependent upon image size *N*.

**Figure 1:**
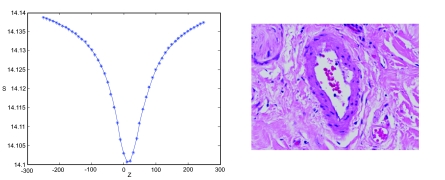
Entropy-based Focus Curve.

A focus curve can be generated by computing the image entropy across the Z-axis for any given microscopic field. As shown in the left panel of Figure 1, the focus curve exhibits an inverse-bell-shape, with the tip of the bell or curve minimum corresponding to the perceptual focal plane, and the flanks, corresponding to out of focus planes, approaching *S*_0_. The same panel illustrates the use of entropy as monotonic indicator of focus. The algorithm directs the robotics and imaging device to acquire a series of images while varying Z positions under a fixed magnification, without horizontal movement. Entropy values are automatically generated.

Due to slight variations in image complexity and sample thickness each microscopic field exhibits a specific set of curve characteristics, however, the overall inverse-bell-shape of each respective curve is generally preserved. Using image entropy as a monotonic indicator of focus, we consider the task of auto-focusing as a function minimization problem while sampling of the *focus curve* is kept to a minimum.

Utilizing a modified golden section search algorithm in one dimension the system reliably delivers an in-focus or nearly-in-focus image each time that a different objective lens is selected. The auto-focus algorithms have been tested using peripheral blood smears, liver biopsies, and breast cancer tissue arrays.

**Figure 2:**
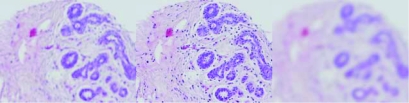
Entropy-based Auto-focus of Breast TMA.

Figure 2 shows three representative images taken from a series acquired while auto-focusing on a breast TMA.

The Distributed Telemicroscopy subsystem (Foran et al. 2000) and Tissue Microarray Imaging and Analysis modules (Chen et al. 2004) were designed and developed in JAVA utilizing a client/server design to maximize portability across computer architectures and operating systems while reducing the computational burden placed upon the server. The Tissue Microarray Repository (TMR) subsystem utilizes the same fundamental design. The improvement of TMR (Tissue Micro-array Repository subsystem) over the previously reported prototype has a few aspects. The TMR is integrated with the Distributed Telemicroscopy(DT) system to better streamline the digitizing and archiving process. The new graphical user interface allow individuals easy access of functions to populate the database with new datasets as well as to manage existing datasets. It also strenghens the administration of the databases. The TMR interface will enable individuals to initiate the *unsupervised* imaging and analysis steps during the course of an active Distributed Telemicroscopy (DT) session. The system automatically performs registration of the specimen, generates the spectral and spatial signature for each disc, and serializes and streams the corresponding images to any number of mirror sites. At this time, the system prompts the user for the name of any correlated clinical reports. A Perl-based program is being developed to automatically process flat-ASCII version of the correlated clinical reports by stripping off salient fields while omitting any patient identifiers such as (Social Security Number, Medical Record Number, Case Accession Number, Address, Phone Number, etc.).

The de-identified data along with the corresponding feature vector housing the image metrics generated during analysis can be transferred to a server-side JAVA application which automatically checks for potential conflicts of new entries, and populates the database with the new set of image metrics and correlated data while inserting an entry into the database to indicate the location of the digitized cancer specimens. The data and identifiers will be developed to meet all HIPAA requirements for sharing data anonymized for research by adapting to the evolving data exchange specification (Berman et al. 2003).

**Figure 3:**
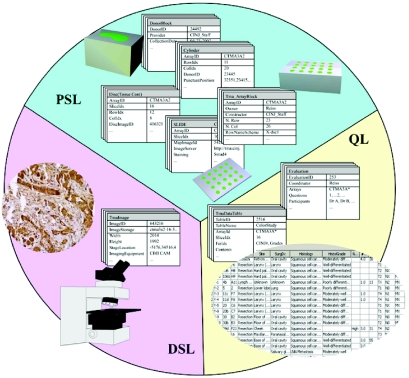
Organization of the Database.

Distributed databases are organized as shown in Figure 3. The physical specimen layer (PSL) of the database records information related to the construction and preparation of the physical TMA sample. The user-friendly graphical interface assists technicians organizing information in the phase of array construction as well as researchers accessing this information. The digital sample layer (DSL) of the database stores archived digital images including the image map and imaged tissue discs (at multiple resolutions). High-resolution images of tissue discs, are broken down into small frames in order to facilitate network access. The third layer of the database, the quantification layer (QL), provides a data structure, which supports automated segmentation and computation of protein expression levels across each disc. We are currently developing and will publish a new TMA metadata specification in XML by combining existing and emerging TMA data exchange specifications (Berman et al. 2003; Berman et al. 2004) with the new image-based feature measurements that are developed during the next phase of the project.

In addition to the algorithmic and software development that is required for analyzing tissue microarrays, reliable tools are also needed to enable individual groups to dynamically acquire and seamlessly share imaged specimens and correlated metadata. In spite of some of the progress that has been made in organizing and standardizing data for exchange, scalable information discovery is quite limited in the absence of global knowledge of naming conventions. This remains a fundamental problem for most applications which must operate in large, decentralized, distributed environments. This is due to the heterogeneous nature and large volume of data and resources, their dynamism and the dynamism of the sharing environment (with nodes joining and leaving). As a result, an information indexing and discovery system has to be efficient, fault-tolerant and self-organizing. In the case of TMA data, the ability of each research group to maintain ownership of their own data while providing access control capabilities is critical.

### A Collaboratory for decentralized TMA data sharing

Recent years have seen increasing interest in peer-to-peer (P2P) information sharing environments. In the P2P computing paradigm, entities at the edges of the network can directly interact as equals (or peers) and share information, services and resources without centralized servers. Key characteristics of these systems include decentralization, self-organization, dynamism and fault-tolerance, which make them naturally scalable and attractive solutions for information storage and discovery applications. As a part of our feasibility studies a prototype TMA collaboratory has been developed utilizing Squid (Schmidt et al. 2004), a P2P information indexing and discovery infrastructure. Squid implements a distributed hash table (DHT) on a self-organizing structured overlay network of peers. Each peer (e.g. research institution) in this system maintains ownership of its data and only publishes (in a controlled manner) metadata describing its TMA and experimental data inventory, which can then be discovered and searched externally. The key innovation is a dimension reducing indexing scheme that effectively maps the multidimensional metadata information space to physical peers. Note that access to TMA data in this system is always controlled by the owner of that data.

**Figure 4:**
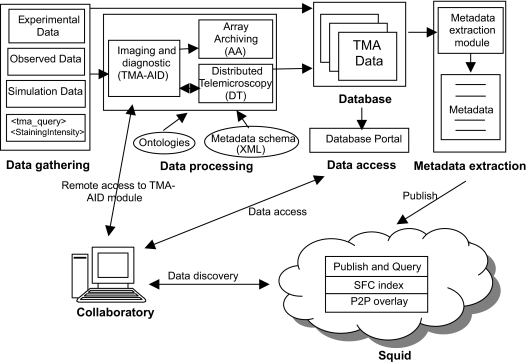
Collaboratory for decentralized information sharing for investigative research and discovery.

A schematic overview of the overall architecture of the prototype collaboratory is presented in Figure 4. The *data gathering* module collects the data that is processed and shared. There are four major types of data: the experimental data, the observed data, the simulation data and the archived data.

The *data processing* module employs vision-based and feature-based techniques to extract relevant data from the images and data associated with the TMA slide (i.e. the TMA-AID system). The extracted data is stored in the database using the Array Archiving (AA) subsystem. The TMA-AID system can be used remotely using the Distributed Telemicroscopy (DT) subsystem. The *data access module* enables remote access to the data stored in the local database.

The *metadata extraction module* extracts information describing the shared data from the local database. The metadata is published in *Squid* P2P storage and discovery system. Finally the *collaboratory GUI* allows users to flexibly search TMA data and metadata in Squid and access it through the Database Portal.

### Squid – A P2P system for information storage and discovery

A key requirement for the TMA collaboratory is the ability to flexibly and efficiently search P2P infrastructure using keywords, partial keywords, wildcards and ranges. Further, the query engine should guarantee that all existing data elements that match a query are found with bounded costs. The Squid query engine supports such complex queries and guarantees that all existing data elements that match a query will be found with bounded costs in terms of number of messages and number of nodes involved. For large systems, for a generic query matching *p%* of the data, the number of nodes with matching data approaches *p%* of all nodes in the system (Schmidt, 2005). Squid uses an optimized search engine based on recursive query refinement, distributing the querying process at multiple nodes in the system (usually the ones that store matching data), and pruning unwanted search paths early.

The architecture of the Squid P2P information retrieval system is based on data-lookup systems (Stoica et al. 2001; Ratnasamy et al. 2001), and essentially implements an Internet-scale distributed hash table (DHT). The key difference is in the way it maps the data to the DHT index. In existing systems this is done using a hashing function that uniformly distributes data to nodes, and as a result data can be retrieved only if its exact identifier is known. In contrast, Squid uses a dimension-reducing mapping called Hilbert Space Filling Curve (SFC) (Sagan, 1994), which is self-similar and recursive, and enables complex queries.

The participating peers in the P2P infrastructure typically run on machines at hospitals, research centers and universities. Specialized software agents at each local site extract metadata from the local database, and publish it in the P2P storage and discovery system. However, rather than storing the data, only references to the data described by the metadata are stored. This behavior is desired because access to data is typically restricted based on access credentials. The Squid P2P infrastructure thus enables global discovery (with desired access control restrictions) of metadata while allowing the peers to maintain ownership and locally control access to their data.

Squid replicates the metadata at multiple nodes, for fault tolerance. Also, to avoid storing metadata for peers that left the system (voluntarily or due to failures), each metadata file indexed has an expiration date associated with it. If the owner of the metadata does not re-publish it before the expiration date, the entry is deleted from the system.

Since peers typically run on dedicated machines, the machines will be likely to be robust and stay alive for longer periods of time, and the P2P system can become quite stable. While this property is not necessary for Squid, which can deal with peers joining, leaving and failing dynamically, it can be exploited to reduce the maintenance costs of the overlay network.

### Extracting and publishing TMA metadata

**Figure 5:**
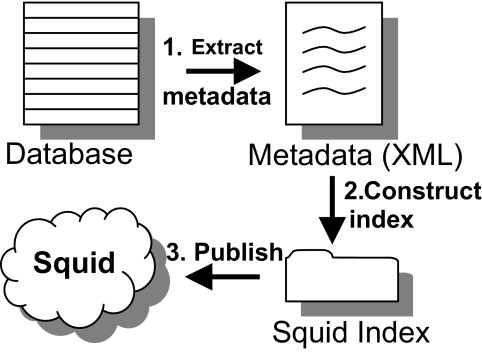
The process of publishing TMA metadata in Squid: the metadata (XML files) is extracted from the database. The metadata is used to publish the XML file and the location of the data in Squid.

**Figure 6:**
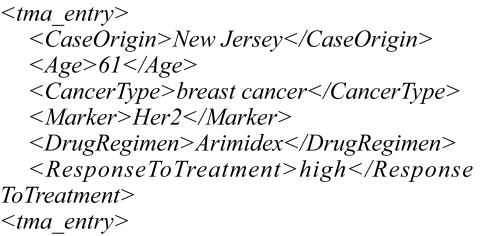
Example of metadata (XML) extracted from the database.

The metadata extraction process is illustrated in Figure 5. The metadata is extracted from the local database by a software agent, who then publishes it into Squid along with references to the data. The agent checks the database for changes at regular intervals, looking for new data. An example of the XML metadata extracted from a database record (a case) is presented in Figure 6.

Note that only the metadata is published in Squid, and not the actual data. In this way, the owners of the data may enforce access control on their data. However, the system does not provide access control on metadata.

To support keyword searches, Squid uses the values of the XML tags as keywords. These values are the keywords used to store a metadata XML file in the overlay, and to search the system. Note that typically only a subset of the xml tags is used to construct an index. The idea is to construct indices using attributes that are frequently used in queries. Multiple indices may be created, using different sets of keys (possibly overlapping).

**Figure 7:**
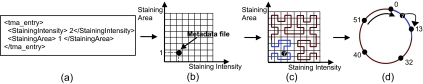
The process of publishing a metadata XML file: (**a**) the metadata XML file; (**b**) the metadata as a point in 2-dimensional space; (**c**) the 2-dimensional point is mapped to the index 7, using Hilbert SFC; (**d**) the metadata is stored in the overlay (an overlay with 5 nodes and an identifier space from 0 to 2^6^–1) at node 13, the successor of the index 7.

The keywords form a multidimensional keyword space, each axis (dimension) representing an XML tag. The metadata files are points in this space and the keywords are the coordinates. The keywords are base-*n* numbers, for example *n* can be 10 for numeric keywords or 26 for English words. An example of a 2-dimensional keyword space is shown in Figure 7(b).

Using the Hilbert SFC (see Figure 7(c) for an example Hilbert SFC in a 2-dimensional space), the multi-dimensional keyword space is mapped to a 1-dimensional index space. A point in the multi-dimensional space is mapped to a point on the SFC. Any range query or query composed of keywords, partial keywords, or wildcards, are mapped to regions in the keyword space and the corresponding clusters (segments on the SFC curve) in the SFC.

The 1-dimensional index space is mapped onto an overlay network of peers. The current implementation of Squid uses the Chord (Stoica et al. 2001) overlay network topology. However, any one-dimensional overlay network can be used within Squid (e.g. Pastry (Rowstron and Druschel, 2001)). Chord is chosen because of its simplicity of design, resilience and performance. Chord implements a Distributed Hash Table (DHT), where the hash table is partitioned and distributed at the peers. The topology of the overlay and the placement of the data are highly structured and tightly controlled, allowing the system to provide access guarantees and bounds.

In Chord each node has a unique identifier ranging from 0 to 2^m^–1. These identifiers are arranged as a circle modulo 2^m^. The data is hashed to numerical identifiers (keys) from the same range. Squid uses the SFC mapping to create the data numerical indices. Each metadata file is mapped, based on its SFC index or key, to the first node whose identifier is equal to or follows the key in the identifier space. This node is called the *successor* of the key. An example of an overlay network with 5 nodes and an identifier space from 0 to 2^6^–1 is shown in Figure 7(d). In this example, the metadata file shown in Figure 7(a) is stored at node 13, since its SFC index is 7, and node 13 is the successor of key 7. Each node maintains information about (at most) *m* neighbors, called *fingers,* in a *finger table.* The finger table is used for efficient routing. Chord essentially implements one operation, lookup(key), which routes the request to the peer responsible for storing the key. When a node receives a query for a key that is not stored locally, it routes the query to the node in its routing table that places the query closest to the destination. In Chord, a data lookup requires O (log N) hops, where N is the number of nodes in the system.

Publishing metadata in Squid consists of the following steps: (1) extract keywords from the metadata XML file; (2) use the SFC-mapping to construct the index from the keywords; and (3) using this index store the metadata at the appropriate node in the overlay. This node is located using the Chord’s lookup mechanism. Figure 7 illustrates the publishing process.

### Querying the system—the squid query engine

The primary function of the query engine is to efficiently process user queries. The expected result of a query is the complete set of data elements that match the user’s query.

**Figure 8:**
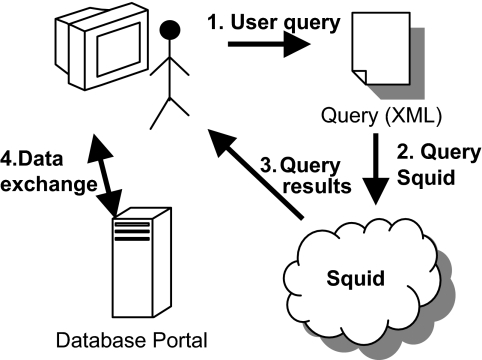
Searching information using Squid.

The system is queried through a friendly graphical user interface (GUI). The user query is presented to Squid as an XML file. Squid parses the document, extracts the user query and resolves it. The results are presented to the user and consist of links to relevant data in databases maintained by hospitals, research centers, etc. The user can then contact the owners of the data to obtain required permission to access the data using the database portal. Note that the access to the data is outside Squid and is subject to the hospital’s (or research center’s) regulations. Figure 8 illustrates this process.

**Figure 9:**
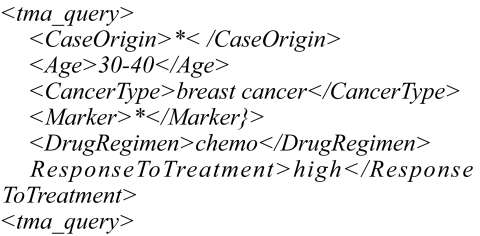
Example of a user query.

The queries can consist of a combination of keywords, partial keywords, or wildcards. An example of a possible user query is presented in Figure 9. In this example, the user is interested in data about patients between 30 and 40, with breast cancer, who are treated with chemo, and have a high response to the treatment. The marker type and the case origin can be anything.

**Figure 10:**
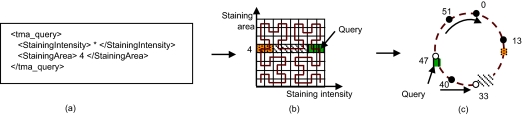
Processing the query (*, 4): (**a**) the query in XML format; (**b**) the query defines a rectangular region in the 2-dimensional keyword space, and 3 clusters (3 segments on the SFC curve); (**c**) the clusters (the solid part of the circle) are stored at nodes 33 and 47, so these nodes will be queried.

If the query consists of complete keywords (no wildcards or ranges) it will be mapped to at most one point in the index space, and the node containing the matching data-element is located using the overlay’s lookup protocol. If the query contains partial keywords, wildcards and/or ranges, the query identifies a region in the keyword space, which corresponds to a set of points in the index space. For example, in Figure 10(a), the query (*, 4) identifies 8 data elements. The index (curve) enters and exits the region three times, defining three segments of the curve or clusters.

Processing a query consists of two steps: Translating the keyword query to relevant clusters of the SFC-based index space, and querying the appropriate nodes in the overlay network for data-elements.

Once the clusters associated with a query are identified, straightforward query processing consists of sending a query message for each cluster, using the lookup mechanism provided by Chord. Figure 10 illustrated the query processing: the query (*, 4) defines a rectangular region in the 2-dimensional keyword space, and identifies three clusters. The clusters are stored into the overlay at nodes 33 and 47, so these two nodes will be queried. The node that initiated the query can not know if a cluster is stored in the network or not, or if multiple clusters are stored at the same node, to make optimizations. The number of clusters can be very high, and sending a message for each cluster is not a scalable solution. The recursive nature of the SFC is used to optimize the query processing. Details about the optimization can be found in (Schmidt and Parashar, 2004).

## An Experimental Evaluation of Squid

This section presents an evaluation of Squid using both simulations and a prototype deployment. Table 1 presents a sample ontology that was used to test Squid during feasibility experiments. During the course of the feasibility experiments we utilized image metrics, which we have already established for integrated staining intensity, effective staining area and effective staining intensity as well as the sample ontology to assess the performance of Squid in locating TMA specimens.

This section presents simulation results that demonstrate the efficiency of the Squid query engine. Two sets of data were used in this evaluation: (1) synthetically generated data, uniformly distributed on the curve, and (2) real data consisting of world-wide publications collected by a search engine.^1^ The objectives of the experiments were to demonstrate that: (1) as the number of nodes in the system grows, the percentage of nodes with matches for a query approaches the percentage of the data matched, and (2) the optimization used by the query engine is successfully reducing the number of clusters that have to be generated for a query, and that the number of extra nodes involved in the process is small.

The performance of Squid is evaluated using a simulator that implements the SFC-based mapping, the overlay network, the load-balancing steps, and the query engine with the query optimizations described earlier.

All queries were issued on a load-balanced system where each node stored the same quantity of data. It was assumed that the system stores only the index (SFC numerical index, keywords, meta-data files and a reference to the data), and not the actual data. Only unique data was used where each data element was described uniquely by keywords, and had a unique SFC index. Queries containing ranges, partial keywords and/or wild-cards were evaluated. The results were grouped by query coverage, i.e. the percentage of data matched, and the average was computed for each group.

Note that the results presented here are independent of the overlay used. Only the nodes involved in query processing were measured. The additional nodes involved in routing the queries are overlay-dependant and are not presented here.

The first set of experiments used a 3-dimensional keyword space composed of 2^24^ cells and systems of sizes 10^3^, 10^4^, 10^5^ and 10^6^ nodes. The system was populated with 2^24^ unique, synthetically generated data elements that completely populated the space and resulted in a uniform distribution.

The second set of experiments was performed using real data. The system was populated with 4x10^5^ unique data elements. The experiments were carried out in a 3-dimensional keyword space, composed of 2^48^ cells, and three system sizes with 10^3^, 10^4^ and 10^5^ nodes.

**Figure 11:**
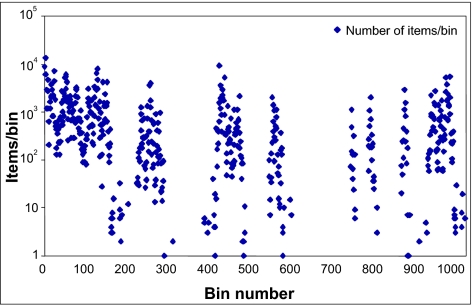
Distribution of data on the Hilbert SFC. The curve has 2^48^ points which are divided into 1000 bins. The *y* axis plots the number of data elements in each bin using a logarithmic scale.

The distribution of the second set of data on the SFC curve is shown in Figure 11. Since the curve has 2^48^ points, it is divided into 1000 bins in the plot. The *x* axis plots the bin number and the *y* axis plots the number of data elements in each bin. As the figure shows, the data is not uniformly distributed. Note that there are empty spaces on the curve, which are primarily due to the fact that the curve is in base 32 (the basic step of recursion is the base-2 Hilbert SFC refined 5 times) while the keywords using the English alphabet are in base 26. However, even if these empty spaces are ignored, the distribution is far from uniform.

**Figure 12:**
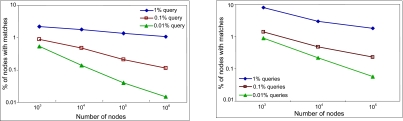
3D data, queries with coverage 1%, 0.1% and 0.01%, plotted using a logarithmic scale on both axes. (**a**) first set of data: uniformly distributed; (**b**) second set of data.

Three classes of queries were used in the evaluation providing coverage of 1%, 0.1% and 0.01% respectively. A query with a coverage of 1% matched 1% of the total data stored in the system. The same set of queries was used for each system size. The results are plotted in Figure 12 using a logarithmic scale on both axes: the *x*-axis plots the size of the system, and the *y*-axis plots the percentage of nodes with data matching the query. The plots show that the percentage of nodes queried decreases as the size of the system increases, and it approaches the percentage of data that the query matches.

### Evaluations of the query engine optimizations

The experiments presented in this section evaluate the optimization strategies used by the query engine. Measurements included the number of clusters generated for a query and the number of nodes involved in resolving a query, with and without the optimization.

**Figure 13:**
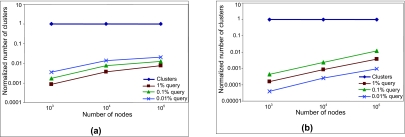
Number of clusters, normalized and plotted on a logarithmic scale. The line at y = 1 represents the clusters that the query defines on the curve. The other lines represent the clusters generated using the optimized query engine for queries with coverage of 1%, 0.1% and 0.01%. (**a**) first set of data, uniformly distributed; (**b**) second set of data.

The number of clusters for queries with coverage of 1%, 0.1% and 0.01% are plotted in Figure 13. Two values were measured for each query; (1) the number of clusters defined by the query on the curve with no optimization, and (2) the number of clusters actually generated in the system when the optimization is used. The values obtained were averaged for each query coverage group and system size. Finally, the results for each query group were normalized and plotted on a logarithmic scale. As Figure 13 shows, the number of clusters that have to be generated is substantially reduced when the optimization is used. For example, for a query with 1% coverage and a system size of 10^4^, only 0.35% of the clusters need to be generated for the first set of data (see Figure 13(a) ), and 0.08% for the second set of data (see Figure 13(b)).

**Figure 14:**
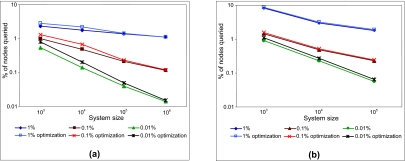
Percentage of nodes queried, with and without the optimization, plotted on a logarithmic scale, for queries with coverage of 1%, 0.1% and 0.01%. (**a**) first set of data, uniformly distributed; (**b**) second set of data.

The number of nodes involved in query resolution is plotted in Figures 14. The results with optimization and without optimization are plotted on the same graph, using a logarithmic scale on each axis. The graphs show that, as described in (Schmidt, 2005), the optimization results in additional nodes being involved in query resolution. However, this overhead induced by the optimization is small (approximately 15% overhead for the first set of data, and 7.5% for the second), when compared to the number of clusters that are pruned.

### Evaluations of the squid prototype

The prototype implementation of Squid is built on Project JXTA, a general-purpose peer-to-peer framework. The overlay network (e.g. Chord) and Squid are implemented as event-driven JXTA services. The prototype system was deployed and evaluated on a Linux cluster consisting of 64 1.6 GHz Pentium IV machines and a 100Mbps Ethernet interconnection. Each of the 64 machines was running a peer in the overlay.

**Figure 15:**
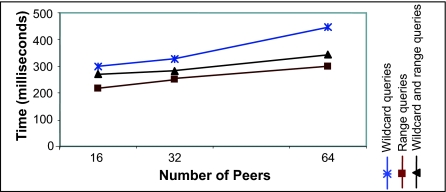
Query processing overhead at a node.

The experiment measured the Squid overhead at a node. Three sets of queries were used, the first containing wildcards, the second containing ranges and the third containing both wildcards and ranges. The query processing overheads at the Squid layer were measured at each node and averaged. The results are plotted in Figure 15. The measured overhead includes times for cluster refinements and subclusters lookup. As Figure 15 shows, the overhead grows slowly and at a much smaller rate than the system size. This demonstrates that Squid can effectively scale to large numbers of nodes while maintaining acceptable query processing times. As expected, the routing times are higher for queries with wildcards as they involve a larger number of clusters and correspondingly larger number of nodes.

Current research includes the development of an image-based metadata specification and combine these new feature measurements with existing and emerging data exchange specifications (Berman et al. 2003; Berman et al. 2004) for cancer TMA’s. These new TMA specifications will be developed and validated in XML in keeping with the existing specifications (Berman et al. 2003; Berman et al. 2004).

## Discussion

Much of the difficulty in rendering consistent evaluation of expression patterns in cancer tissue microarrays is due to subjective impressions of observers and it has been shown that when characterizations are based upon computer-aided analysis, objectivity, reproducibility and sensitivity improve considerably. Advanced imaging and computational tools could potentially enable investigators to detect and track subtle changes in measurable parameters leading to the discovery of novel prognostic clues, which may not be apparent by human inspection alone. In modern cancer research large-scale projects involving tissue microarrays will inevitably involve multi-institutional efforts in which investigators and resources are spread out over multiple campuses, cities, and states. Using modern discovery tools it is now possible to enable individuals to automatically detect, locate and share experimental results and specimens from de-centralized sources distributed across the cancer research community. The distinguishing characteristics of the prototype system that we have described is that it can self-organize and through the implementation of a dimension reducing indexing scheme is able to effectively map multidimensional metadata information space to physical peers. Each peer (e.g. research institution) in this system maintains ownership of its own data and only publishes (in a controlled manner) metadata describing its data, which can then be discovered and searched externally. In the next phase of the project we will determine a definitive set of image-based feature measurements which are shown through robust statistical methods to best represent the under-lying pathology of the cancer tissues under study (head, neck, breast) and incorporate them with emerging TMA metadata standards. As part of our initial feasibility studies the TMA and Squid software was deployed at strategic sites at the University of Medicine & Dentistry of New Jersey (UMDNJ), Rutgers University (RU), and The Cancer Institute of New Jersey. In the next phase of experiments we plan to deploy and evaluate the TMA/Squid systems at strategic sites throughout UMDNJ, RU, CINJ and the University of Pennsylvania (UPenn), Arizona Cancer Center, and Penn State Cancer Institute.

## Figures and Tables

**Table 1:** Sample TMA ontology.

Cancer Type	Marker	Response	Drug Regimen
adenocarcinoma of the lung	ASCT2	high	Arimidex
bladder cancer	bcl-6	intermediate	Bexxar
breast cancer	CD10	low	chemo
bronchioloalveolar carcinoma of the lung	Cd44	normal	Eloxatin
colorectal carcinoma	COX-2		Faslodex
Diffuse Large B Cell Lymphoma(GC)	Fas		Gefitninib
Diffuse Large B Cell Lymphoma(ABC)	Her2		Trastuzumab
non-small cell lung cancer	hMLH1		CPT-11
prostate adenocarcinoma	Ki-67		Bruceantin
small cell lung cancer	MMX-9		Letrozole
	p53		
	PTEN		
	Stat3		
	TTF-1		
	Tyr705		

1 The data used in this experiment was obtained from Citeseer (http://citeseer.ist.psu.edu/).
